# Issues of cost-benefit and cost-effectiveness for simulation in health professions education

**DOI:** 10.1186/s41077-016-0020-3

**Published:** 2016-05-17

**Authors:** Stephen Maloney, Terry Haines

**Affiliations:** 1 Department of Physiotherapy, Monash University, Melbourne, Australia; 2 Medical Education Research and Quality (MERQ) Unit, Department of Epidemiology and Preventive Medicine, Monash University, Melbourne, Australia; 3 Allied Health Research Unit, Monash Health, Melbourne, Australia

**Keywords:** Simulation, Costs, Benefits, Effectiveness, Value, Market

## Abstract

**Background:**

Simulation education can be costly—however, costs need to be considered against what you get in return to determine whether these costs are justified. Unfortunately in simulation education, evaluations that yield information about the return on investment are scarce. An economic evaluation provides a comparison of value. In short—what is it that is being obtained, what do you need to give up to get it, and how does that compare to what you get with the next best alternative? When educators are equipped with this knowledge, they will be better informed to know the place that simulation-based learning approaches should take in optimal course structures.

**Main body:**

This article provides an overview of the costs and consequences associated with simulation in healthcare education. It provides an outline of the benefits of using economic evaluations to inform decision-making by educators and clinicians concerning the most appropriate educational approaches. It also provides guidance for educational researchers interested in investigating the cost and value of their innovations.

**Conclusion:**

Measures of cost and value in simulation are required to provide information about the viability and sustainability of simulation education, enabling simulation education in health care to demonstrate its worth.

## Background

Advancement in health professional workforce training through the use of simulation education is an area of research and practice that has grown rapidly over the last decade [1]. With simulation education permeating into regular teaching learning and assessment practices, it is now commonplace in health professional programs around the world [2].

The training of health workforce professionals in many countries is heavily subsidised through the public purse, with national yearly expenditure entering the $USD billions [3]. In these contexts, there is a need to select efficient educational methodologies to ensure that maximum value is being returned to society through this investment. Even with direct payment by students in full, there is need for universities to identify efficient educational methodologies so that their courses can remain competitive in a global marketplace.

As described in the definition by Professor David Gaba, at its heart, ‘simulation is a technique to replace or amplify real experiences with guided experiences, often immersive in nature, that evoke or replicate aspects of the real world in a fully interactive fashion’ [4]. Simulation is a rapidly developing approach used to substitute for clinical experiences [5, 6], but can be an expensive modality for teaching and learning. The price tag for an Objective Structured Clinical Examination for 185 students in a UK-based medical school was greater than 65000 GBP or 355 GBP per student [7]. Such expenses are sobering; however, no expense can be put in context without considering what you get in return. Unfortunately in simulation education, evaluations that yield information about the return on investment are scarce [2]. Further, simulation can vary in most of the components that create the learning experience. Simulation can be high cost, or low cost, depending on the methods, technology, and fidelity of the simulation. It can use peers and confederates, professional actors or mannequins, be conducted live or in web-based ‘virtual’ environments. Skills to be taught can range from specific procedural skills [8], such as cannula insertion, or based around high level communication skills [9], such as gaining consent for organ donation [10]. With many competing approaches available, and limited resources in which to implement them, educators must be judicious in their selection of each approach. To do so, educators require an understanding of both the costs and consequences associated with each. This paper aims to highlight the relevant costs and consequences that need to be examined when considering the use of simulation education in health care.

## What is an economic evaluation?

An economic evaluation provides a comparison of value, revolving around the concept that we have finite resources and that you can only spend each dollar once. In short—what is it that is being obtained, what do you need to give up to get it, and how does that compare to what you get with the next best alternative? Drummond’s checklist of cost and consequences in healthcare interventions and programs provides a more in depth description of its elements [11], covering aspects such as did the study examine both costs and effects of the intervention? Did the study involve a comparison of alternatives? Was a viewpoint for the analysis stated and was the study placed in any particular decision-making context? Were costs and consequences adjusted for differential timing? Were the additional (incremental) costs generated by one alternative over another compared to the additional effects, benefits or utilities generated?

We unwittingly undertake economic evaluations regularly in our everyday lives, such as when we decide what we will buy within an allocated budget at a grocery market. Will my family gain more benefit from purchasing a packet of chocolate biscuits compared to spending the same amount on a bunch of bananas? The same decision-making processes used to address these questions can be applied in different contexts. In the field of health economics, we are interested in the health service personnel, consumables (including pharmaceuticals) and capital (e.g. equipment, buildings) that may need to be purchased for the betterment of the health of a community. The different processes used to combine these ingredients into a ‘health service’ are also of great interest. These concepts are no different when they are applied to educational interventions, though there are some important differences. We now highlight these similarities and differences, with particular reference to how they can be applied to evaluate the economic efficiency of simulated learning environments.

## Measurement of costs

A starting point for any economic analysis is the attainment of costs. Fixed costs are costs that remain constant regardless of how many goods are being produced [12]. Examples of fixed costs within simulation may include the development of scripts or the purchase of mannequins. Variable costs are costs that vary with the level of output, i.e. that change with the number of students going through the simulation education. Variable costs in simulation may include printing costs and consumables, the number of standardised patients required, staffing costs and space charges.

It is important to consider all the costs relevant to a particular stakeholder or viewpoint chosen for the analysis, as this may impact on the evaluation results. Costs can be incurred differently for different stakeholders. For example, Haines et al. conducted a cost-minimisation analysis of interprofessional clinics versus traditional profession-specific clinical education in hospitals. Their analysis revealed that the interprofessional clinic, when contrasted to traditional hospital-based placements, produced an additional cost to the university of $289 per student day; however, a saving of $49 was found for the state government viewpoint, a saving of $66 for the Commonwealth Government, while society overall incurred an additional expense of $175 per student day [13]. Interestingly, simulation education often crosses both the education sector and the healthcare sector, creating potential for differing amounts of costs and outcomes to be borne by these stakeholders from the one economic evaluation.

A misconception in determining the costs involved in an educational intervention is including the transition costs to enable the trial or comparison to be made. As these costs have already been invested, they no longer have an opportunity cost as they cannot be used for another purpose. They are referred to as being ‘sunk’ costs. The up-front cost of creating a ‘simulation centre’ should normally not be included in an economic evaluation of a program that uses these facilities. Rather, a rental charge for using these facilities based upon ‘market rate’ charges should normally be used. The key exception to this would be if one were to conduct a particular form of economic evaluation known as a ‘break-even analysis’. Here, the focus of the evaluation may be to identify the number of students that a hospital or university would need to educate through a new, proposed simulation centre in order for the revenue raised from this activity to equal the cost of building and running this centre.

## Consequences

The consequences of simulation education relevant in an economic evaluation can occur in any level of Kirkpatrick’s hierarchy. For example, we might hypothesise that the appropriate use of quality simulation practices will more deeply engage the learner, enhance their motivation and improve their clinical performance, leading to behaviour changes. In turn, positive changes in clinical practice may lead to better health outcomes [1]. All of these consequences can be relevant to the economic analyses, depending on the research question and outcome of interest. It should be remembered that different stakeholders may obtain different benefits. For example, a student may gain in their understanding and competency, and the health service may obtain a clinical benefit such as improved adherence to best-practice, and there may also be an organizational benefit, such as improved efficiency of patient flow through emergency. A consequence can be financial in nature or it may be less tangible. A financial benefit in simulation education may be the cost averted through students making fewer errors in real patient situations. Benefits like these that can be monetized can be included in the cost side of a cost-effectiveness analysis. Less tangible benefits may include improved patient-centred care, increased empathy, and a greater understanding of the clinical relevance of the skills and knowledge being taught, influencing future learning experiences [1].

It is interesting to consider if it is possible to avert costs in education. For example, the consequences of improved teaching and learning practices through simulation education may be that fewer students fail to gain the appropriate knowledge and understanding and skills required to pass an academic unit. This will have a direct impact on the costs born from the student’s perspective in terms of student fees, and ergo, the societal perspective. It is also foreseeable that a reduction in the number of underperforming students may reduce the University’s costs in supporting underperforming students and resourcing supplementary clinical placements. It may impact on the health service and societal costs through improved clinical performance and career longevity [14].

A common stumbling block for designing economic evaluations is determining how to value and contrast a range of consequences experienced by different stakeholders. For example, how can you compare an education approach that enhances the number of students educated versus one that enhances the quality of learning outcomes for a smaller number of students? One approach proposed has been to combine these metrics into a ‘quality-adjusted student educated metric’ which is analogous to the quality-adjusted life year approach used in many health economics evaluations [15]. Other combinations of outcomes may not be so easily resolved, for example, how do you value a negative learning experience for a student, who goes on to provide improved patient care? One solution may be to make one particular outcome within your analysis primary relevant to the chosen context and base the study conclusions primarily on this [16]. Another method that can be used is to convert relevant consequences into monetary terms [17]. For example, Maloney et al. investigated willingness to pay of health professionals for a web-based education program that they had just completed. The health professional participants nominated values based on their perception of the educational benefits received to their employment and careers [18]. In some contexts, researchers may use empirically determined values to monetarize the consequences of a simulated education training program. For example, a simulated education program that aims to teach skills that would enable a reduction in length of stay in hospital for a particular patient group could use a specific region’s funding formula to calculate the value of any change in length of stay [19, 20].

## What outcome would an economic evaluation produce?

**Fig. 1:**
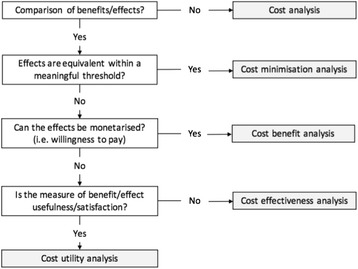
Flowchart of the relationship between benefits/effects and the available type of analysis

As a general guide, if a trial is attempting to demonstrate that a simulation education approach is superior to an alternate approach (or a ‘do nothing’ control), then a cost-effectiveness analysis should be undertaken to produce an incremental cost-effectiveness ratio such as the ‘cost ($) per additional quality-adjusted student educated’. If the consequences of interest can be monetarized is some manner, then a cost-benefit analysis could also be conducted to produce an estimate of net ($) benefit per student educated. If the trial was designed to test whether a simulation education approach produces equivalent outcomes to an alternate approach of greater or lesser cost, and this was found to be the case, then cost-minimisation analysis could be used to produce an estimate of the cost ($) difference per student educated. If an educator is interested in understanding how the cost of educating students using a simulation approach changes with an increasing number of students in the class, then a break-even analysis could be conducted to identify the number of students required to be educated in the class before the costs ($) of using this approach are outweighed by the ($) benefits. Various break-even points can then be compared between competing education methods. Each of these economic evaluation approaches offer further information to the decision maker about the viability and sustainability of the simulation education, enabling simulation education in health care to demonstrate its relative worth compared to other approaches for delivering education. A description of the common types of analysis relevant to simulation education is presented in Table 1, and the relationship between the benefits/effects and the available analysis in Fig. 1.

**Table 1:** Common types of analysis relevant to simulation education

Analysis	Description of measurement of benefits/effects	Hypothetical example applied to simulation
Cost minimisation analysis (CMA)	A comparison of costs when the effects are considered equal in all respects	Measurement of the simulation education method versus an alternative education method produced equivalent learning outcomes within a meaningful threshold; however, the simulation method is less costly.
Cost-effectiveness analysis (CEA)	Benefits/effects are measured in natural units (e.g. students educated)	Measurement of the simulation education method resulted in less clinical errors by the learner than the alternative education method.
Cost utility analysis (CUA)	Benefits/effects measured in ‘utility’ units—e.g. a measure of satisfaction derived from consumption/attainment of benefit	Measurement of the simulation education method resulted in higher levels of patient satisfaction with their care compared to the alternative education method.
Cost benefit analysis (CBA)	Benefits monetized	The simulation education method was measured to be of higher value (willingness to pay) by the learners when compared to the alternative education method.

## Why are not economic evaluations in this field more common?

There are a number of possible reasons why economic evaluations within simulation education, and education more broadly, are less common than in other disciplines and fields of research [21]. Economic evaluations are largely based in a quantitative research paradigm, whereas medical education and educational research is emerging from a time predominantly focused on qualitative research methods [22, 23]. There are a diverse range of stakeholders involved in simulation education, i.e. the learner, patient, health service, administrators, simulation centres, educational institutions and industry. The broader stakeholder base makes the design of an evaluation inherently more complicated. Frameworks for how to do this have only recently been developed [15]. Simulation education in health care encompasses a myriad of variations on available methodologies, making the generalizability of the findings difficult. Staff whose careers and livelihoods are based on running simulation centres may feel these would be threatened if an ‘unfavourable’ economic evaluation were to be published. However, this disincentive to conducting an economic evaluation may be turned into an incentive to conduct an economic evaluation if funding bodies say they will no longer support simulation centres until they have demonstrated their cost-effectiveness.

## Future directions

There is great need to know whether simulated learning approaches are efficient. Even if simulation is effective, it does not mean that the cost of providing it is justified [24]. This would begin with simulation education researchers describing the costs of competing approaches in conjunction with measurement of the benefits and effects. Economic evaluations are only as strong as our confidence in the measure of benefits and effects—caution should be taken in forming strong conclusions based upon studies that have employed study designs at high risk of bias. There are now several trials investigating the effectiveness of simulated learning approaches [5, 6, 25]. These studies could be used to inform economic evaluation modeling studies [26]. However, future research should pre-plan to investigate the efficiency of simulated learning approaches so that economic evaluations can be conducted concurrently alongside these trials. This might prevent findings that may support the cost-effectiveness of simulation education being applied to low level study designs [20] and encourage analyses applied to randomised controlled trials [27]. We need to build on the work of Walsh et al. who provide worked examples different cost-analyses and their usage in the context of medical education [28] and that of Tolgaard et al. who provide a model for applying CEA to a simulation intervention [29]. The leadership of these and other educational researchers and economists could be extended through the development of a best practice reporting guideline, along with conventions for conducting a meta-analysis across this field. Perhaps of greatest importance is the need to plan for how the many different permutations and combinations of techniques that make up the family of simulation-based education will be pooled and sub-grouped. This may require an agreed taxonomy of simulation-based education approaches to be developed and with detailed reporting of these interventions in published papers to allow proper classification [30].

## Conclusion

Advancement in health professional workforce training through the use of simulation education is expanding. However, resources available to provide education are finite and it is necessary to identify education delivery methods that can maximize learning outcomes within these funding constraints. Innovations in education that demonstrate effectiveness need to be subjected to economic evaluations to ensure that they are efficiently relative to the next best approach available. This needs to be demonstrated across a range of contexts and fields to ensure that interactions between these factors and efficiency outcomes are understood. When educators are equipped with this knowledge, they will be better informed to know the place that simulation-based learning approaches should take in optimal course structures.

## References

[1] Nestel D, MB (2015). Simulated patient methodology: theory, evidence and practice.

[2] Zendejas B, Wang AT, Brydges R, Hamstra SJ, Cook DA (2013). Cost: the missing outcome in simulation-based medical education research: a systematic review. Surgery.

[3] Walsh K, Reeves S, Maloney S (2014). Exploring issues of cost and value in professional and interprofessional education. J Interprof Care.

[4] Gaba DM (2004). The future vision of simulation in health care. Qual Saf Health Care.

[5] Blackstock F, Watson K, Morris N, Jones A, Wright A, McMeeken J (2013). Simulation can contribute part of cardiorespiratory physiotherapy clinical education: two randomised trials. Simul Healthc.

[6] Watson K, Wright A, Morris N, McMeeken J, Rivett D, Blackstock F (2012). Can simulation replace part of clinical time? Two parallel randomised controlled trials. Med Educ.

[7] Brown C, Ross S, Cleland J, Walsh K. Money makes the (medical assessment) world go round: the cost of components of a summative final year Objective Structured Clinical Examination (OSCE). Med Teach. 2015;29:1-7. 10.3109/0142159X.2015.1033389 25923233

[8] Nestel D, Groom J, Eikeland-Husebo S, O'Donnell JM (2011). Simulation for learning and teaching procedural skills: the state of the science. Simul Healthc.

[9] Bearman M, O'Brien R, Anthony A, Civil I, Flanagan B, Jolly B (2012). Learning surgical communication, leadership and teamwork through simulation. J Surg Educ.

[10] McCann E, Gatward J. Introducing simulation training to improve the organ donation conversation. Suppl Transplant. 2013;96(10S):433.

[11] Cartwright WS (1999). Methods for the economic evaluation of health care programmes, second edition. By Michael F. Drummond, Bernie O'Brien, Greg L. Stoddart, George W. Torrance. Oxford: Oxford University Press, 1997. J Ment Health Policy Econ.

[12] Walsh K (2011). Defining and costing educational interventions. Med Educ.

[13] Haines TP, Kent F, Keating JL (2014). Interprofessional student clinics: an economic evaluation of collaborative clinical placement education. J Interprof Care.

[14] McAllister M, McKinnon J (2009). The importance of teaching and learning resilience in the health disciplines: a critical review of the literature. Nurse Educ Today.

[15] Haines T, Isles R, Jones A, Jull G (2011). Economic consequences in clinical education [online]. Focus on Health Professional Education: A Multi-disciplinary Journal.

[16] Assmann SF, Pocock SJ, Enos LE, Kasten LE (2000). Subgroup analysis and other (mis)uses of baseline data in clinical trials. Lancet.

[17] Haines T, Brown C, Morrison J (2008). Public provision of four-wheeled walkers: contingent valuation study of economic benefit. Australas J Ageing.

[18] Maloney S, Haas R, Keating JL, Molloy E, Jolly B, Sims J (2012). Breakeven, cost benefit, cost effectiveness, and willingness to pay for web-based versus face-to-face education delivery for health professionals. J Med Internet Res.

[19] Authority IHP. National Weighted Activity Unit (NWAU) Calculators 2015-16: Independent Hospital Pricing Authority; 2015 [Australian Governmnent Publication]. Available from: https://www.ihpa.gov.au/publications/national-weighted-activity-unit-nwau-calculators-2015-16.

[20] Cohen ER, Feinglass J, Barsuk JH, Barnard C, O'Donnell A, McGaghie WC (2010). Cost savings from reduced catheter-related bloodstream infection after simulation-based education for residents in a medical intensive care unit. Simul Healthc.

[21] Walsh K (2013). Cost and value in healthcare professional education—why the slow pace of change?. Am J Pharm Educ.

[22] Ilic D, Maloney S (2014). Methods of teaching medical trainees evidence based medicine: a systematic review. Med Educ.

[23] Tavakol M, Sandars J (2014). Quantitative and qualitative methods in medical education research: AMEE Guide No 90: part I. Med Teach.

[24] Haines T, O'Brien L, McDermott F, Markham D, Mitchell D, Watterson D (2014). A novel research design can aid disinvestment from existing health technologies with uncertain effectiveness, cost-effectiveness, and/or safety. J Clin Epidemiol.

[25] Steadman RH, Coates WC, Huang YM, Matevosian R, Larmon BR, McCullough L (2006). Simulation-based training is superior to problem-based learning for the acquisition of critical assessment and management skills. Crit Care Med.

[26] Maloney S, Nicklen P, Rivers G, Foo J, Ooi YY, Reeves S (2015). A cost-effectiveness analysis of blended versus face-to-face delivery of evidence-based medicine to medical students. J Med Internet Res.

[27] Isaranuwatchai W, Brydges R, Carnahan H, Backstein D, Dubrowski A (2014). Comparing the cost-effectiveness of simulation modalities: a case study of peripheral intravenous catheterization training. Adv Health Sci Educ Theory Pract.

[28] Walsh K, Levin H, Jaye P, Gazzard J (2013). Cost analyses approaches in medical education: there are no simple solutions. Med Educ.

[29] Tolsgaard MG, Tabor A, Madsen ME, Wulff CB, Dyre L, Ringsted C (2015). Linking quality of care and training costs: cost-effectiveness in health professions education. Med Educ.

[30] Hoffmann TC, Glasziou PP, Boutron I, Milne R, Perera R, Moher D (2014). Better reporting of interventions: template for intervention description and replication (TIDieR) checklist and guide. BMJ.

